# Is Physiological Performance a Good Predictor for Fitness? Insights from an Invasive Plant Species

**DOI:** 10.1371/journal.pone.0076432

**Published:** 2013-10-25

**Authors:** Marco A. Molina-Montenegro, Cristian Salgado-Luarte, Rómulo Oses, Cristian Torres-Díaz

**Affiliations:** 1 Centro de Estudios Avanzado en Zonas Áridas (CEAZA), Facultad de Ciencias del Mar, Universidad Católica del Norte, Coquimbo, Chile; 2 Departamento de Biología, Universidad de la Serena, La Serena, Chile; 3 Laboratorio de Genómica y Biodiversidad (LGB), Departamento de Ciencias Naturales, Universidad del Bío-Bío, Chillán, Chile; University of Innsbruck, Austria

## Abstract

Is physiological performance a suitable proxy of fitness in plants? Although, several studies have been conducted to measure some fitness-related traits and physiological performance, direct assessments are seldom found in the literature. Here, we assessed the physiology-fitness relationship using second-generation individuals of the invasive plant species *Taraxacum officinale* from 17 localities distributed in five continents. Specifically, we tested if i) the maximum quantum yield is a good predictor for seed-output ii) whether this physiology-fitness relationship can be modified by environmental heterogeneity, and iii) if this relationship has an adaptive consequence for *T. officinale* individuals from different localities. Overall, we found a significant positive relationship between the maximum quantum yield and fitness for all localities evaluated, but this relationship decreased in *T. officinale* individuals from localities with greater environmental heterogeneity. Finally, we found that those individuals from localities where environmental conditions are highly seasonal performed better under heterogeneous environmental conditions. Contrarily, under homogeneous controlled conditions, those individuals from localities with low environmental seasonality performed much better. In conclusion, our results suggest that the maximum quantum yield seem to be good predictors for plant fitness. We suggest that rapid measurements, such as those obtained from the maximum quantum yield, could provide a straightforward proxy of individual’s fitness in changing environments.

## Introduction

Plant biologists have long been fascinated by the question if photosynthetic rate should increase carbon gain leading to an increase in fitness through higher survivorship or fecundity (sensu [1]). For example, several ecophysiological studies have evaluated plant performance by measuring functional traits related to photosynthesis under different stressors [2-4]. Since photosynthesis is very sensitive to abiotic and biotic stress [5], any individuals exposed to a stressful scenario may show diminished photosynthetic rates.

A higher photosynthesis rate leads to increased resource uptake that can be allocated to different plant functions, thus improving growth, fecundity, and survival [1,6]. Relative growth rate can be modeled as a product of net assimilation rate, of which photosynthesis is one of the major components. Because variation in this component will contribute to relative growth rate and its effects can vary in magnitude [7], a higher photosynthetic rate will confer a higher growth rate. Although, these models do not extend to estimates of fitness such as survival or fecundity, increased assimilation rates should confer an increased fitness. In natural populations, plant growth and other morphological traits have positive correlations with components of fitness [8-10]. Nonetheless, direct relationships between physiological traits and fitness are seldom found in the literature (but see, 1).

Chlorophyll fluorescence parameters (e.g., maximum quantum yield) have been used to obtain qualitative and quantitative information about the photosynthesis process [11-14]. Fluorescence estimates the maximum operating quantum efficiency of electron transport through photosystem II (PSII) in leaves and reflects the internal status of the photosynthetic machinery [13,15,16]. Hence, changes in chlorophyll fluorescence could be interpreted as changes in photosynthetic activity. Because changes in these traits are very susceptible to environmental conditions, it has been widely employed to detect the effects of environmental changes and stress factors on physiological performance [2,17,18]. Furthermore, and based on the correlation between quantum efficiency (estimated by chlorophyll fluorescence) and rate of carbon exchange, some authors have argued that chlorophyll fluorescence parameters are suitable proxies of growth and even fitness (see, 18,19). Despite the extensive knowledge about the use of chlorophyll fluorescence as indicator of plant stress [1,4,19,20], there is no consensus regarding its usefulness as an estimator of whole plant fitness. For example, Arntz et al. (2000) [1] showed that a lower photosynthetic rate decreased the fecundity and survival in different family lines of *Amaranthus hybridus*. On the other hand, Figueroa et al. (1997) [19] used 11 Mediterranean plant species to show that low values of the maximum quantum yield are correlated with high mortality when plants experienced high stress induced by drought. Nonetheless, and to the extent of our knowledge, there are no studies to date that explicitly evaluate the cause-effect relationship between chlorophyll fluorescence and plant fitness.

Plant fitness –defined as the relative reproductive success of a genotype as measured by survival, fecundity or other life history parameters [21]- is highly dependent on a series factors such as environmental stress, life cycle, and breeding system. As pointed out by Valladares (1999) [22] the Darwinian fitness concept is easy to understand, however, it can be extremely difficult to measure directly and it can be only measured in relative terms. Hence, plant fitness is estimated through indirect parameters such as plant size, adult longevity, or growth, which are correlated to fitness, but also to plasticity. We performed a controlled experiment with individuals from the invasive species *Taraxacum officinale* Weber ex F. H. Wigg (Dandelion) from five continents as plant model because is an apomictic plant. It reproduces through unfertilized ovules that are genetically identical to the mother plant [23]. In this way, we were able to evaluate the extent of the relationship between physiological performance and fitness and to properly isolate if a physiology-fitness relationship between different genotypes has an adaptive basis. Specifically, we tested the prediction that photochemical efficiency can be a good predictor of plant fitness by using second generation (F_2_) individuals of *T. officinale* as to ask whether: (i) Are higher photochemical efficiency values correlated with higher female fitness, measured as seed-output?, (ii) Does environmental heterogeneity modify the strength of the relationship between physiological performance and female fitness?, and (iii) Has the variation in the physiology-fitness relationship any adaptive relevance under different abiotic scenarios?

## Materials and Methods

### Seed collection and growth conditions

**Figure 1:**
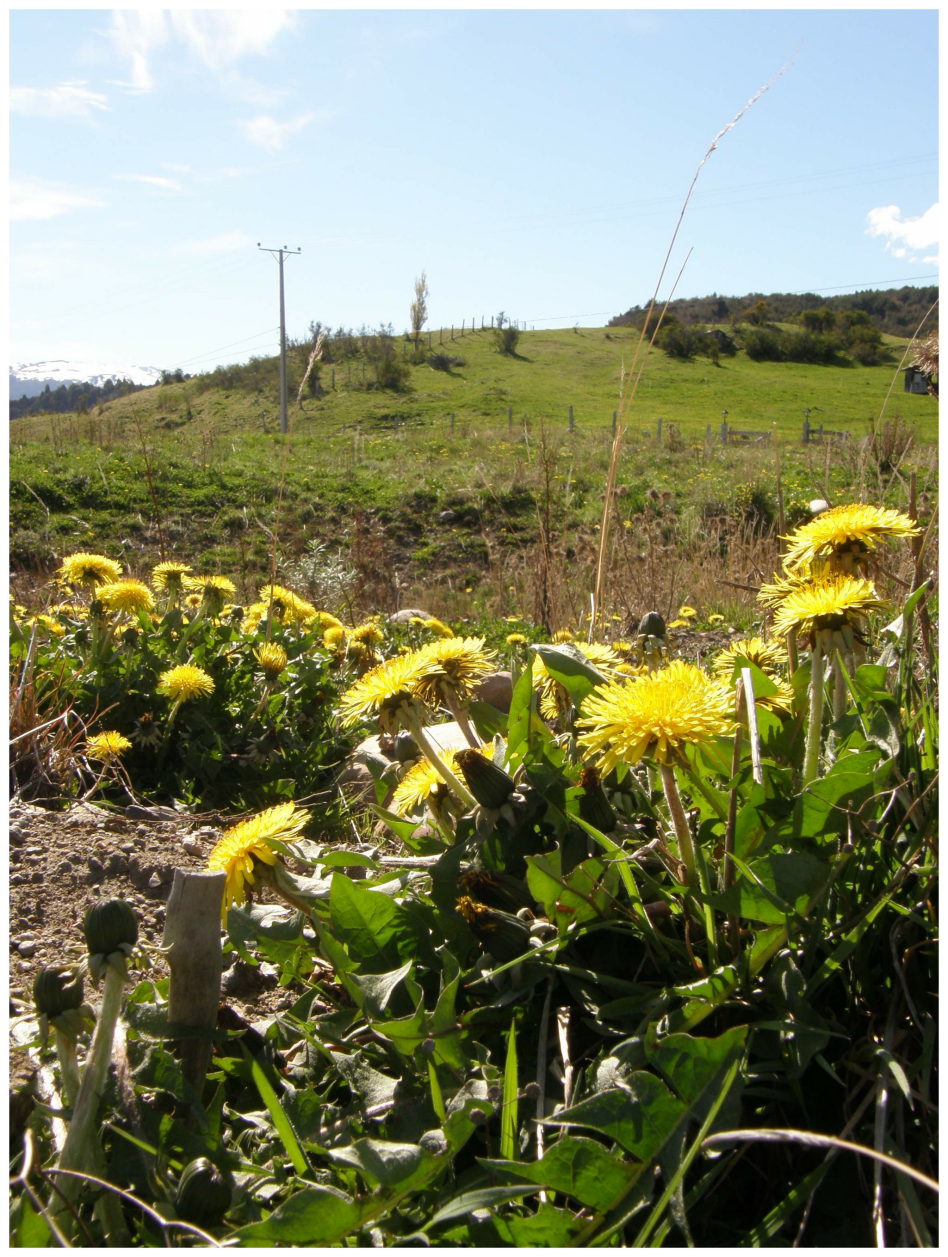
*Taraxacum officinale* population growing in the field in southern Chile. Photographed by Alejandra Lafon and Marco A. Molina-Montenegro in March 2009.

*Taraxacum officinale* (dandelion) is a perennial herb with leaves in rosettes at the soil level, showy yellow heads, and anemochorous seeds (Figure 1). *T. officinale* is originated in central Europe and is considered one of the most aggressive invasive plants around the world [24]. *T. officinale* blooms year-round and is found growing in disturbed and undisturbed sites across a wide altitudinal and latitudinal gradient [25].

Seeds of *T. officinale* were collected in 17 localities from five continents (Table 1). Within each locality a small number of seeds (four to five) per forty to forty-five individual plants were collected. This material provided the initial pool of seeds. Seeds from all localities were germinated in a room at 24 ± 2 °C on wet filter paper in Petri dishes and planted in 500-mL plastic pots filled with potting soil. First generation plants (F_1_) were generated from this initial seed pool and were grown in a greenhouse at Universidad de Concepción, Concepción, Chile (36°48´ S, 73°03´ W) under natural conditions of light and temperature (1300 µmol m^-2^s^-1^ ± 50 and 22 ± 2 °C, respectively). All plants were watered daily with 75 ml of tap water. After five months these plants produced the achenes that were used to obtain experimental plants (F_2_).

**Table 1:** Populations indicating the geographic coordinate where *Taraxacum officinale* seeds were collected.

**Continent**	**Locality**	**Latitude**	**Longitude**	**Seed-output**	**Fv / Fm**	**CV-pp**	**r^2^**
Africa	Cape Town	-33.54	-18.24	0.929	0.752	63.6	0.78
Europe	Amsterdam	52.24	-4.56	0.906	0.740	25.0	0.75
Europe	Barcelona	41.29	2.05	0.922	0.765	33.1	0.74
Europe	Bruselas	50.51	-4.16	0.913	0.752	29.5	0.80
Europe	Estambul	41	-28.58	0.923	0.754	51.9	0.73
Europe	Londres	51.26	0.11	0.911	0.755	22.5	0.74
Europe	París	49.03	-2.15	0.903	0.748	32.9	0.85
North America	San Francisco	37.48	122.28	0.930	0.757	107.1	0.66
North America	Wisconsin	43.5	88.49	0.911	0.746	60.5	0.74
Oceania	Wellington	-41.18	-172.46	0.961	0.753	23.2	0.84
South America	Buenos Aires	-34.48	58.32	0.967	0.784	22.5	0.77
South America	Cartagena	10.23	75.31	0.965	0.789	45.5	0.79
South America	Concepción	-36.48	73.02	0.945	0.776	96.9	0.63
South America	Manta	0.56	80.41	0.961	0.789	85.9	0.73
South America	Punta Arenas	-53.09	70.53	0.912	0.754	22.4	0.84
South America	São Paulo	-23.33	46.37	0.959	0.792	47.1	0.82
South America	Trujillo	-8.06	79.02	0.958	0.790	195.4	0.46

Are shown the maternal fitness (seed-output), maximum quantum yield (physiological performance), and the adjusted R-square obtained from Pearson correlations among both variables.

No specific permits were required for seed collection in the localities sampled in this study and confirm that all populations are not privately-owned or protected in any way. Additionally, we confirm that the field studies did not involve endangered or protected species.

### Experiment of environmental variation

Second generation seedlings from all localities were planted in 500-mL plastic pots filled with potting soil, and transferred to greenhouse. Two watering regimes were applied to individuals from all populations. In the homogeneous environmental regime, plants received 50 ml of water every day (350 ml per week). Plants in the heterogeneous environmental regime also received a total of 350 ml of water, but watering was randomly distributed during the week, with a minimum and maximum amount of 25 and 100 ml of water irrigation, respectively. The full experiment resulted in 12 replicates x 2 watering scenarios x 17 localities = 408 pots in total. Pot position within the greenhouse was changed every five days and interpot distances were sufficient to prevent mutual shading. Plants were supplemented with 0.2 g l^-1^ of Phostrogen® (Solaris, N-P-K, 14:10:27) once every 15 d. Experimental treatments lasted for 150 days and then we measured maximum quantum yield and seed-output as proxies of physiological performance and fitness traits, respectively.

### Measurements of physiological performance and seed-output

We measured physiological performance (maximum quantum yield) at the end of the anthesis at room temperature using a pulse-amplitude modulated fluorometer (FMS 2, Hansatech, Instruments Ltd, Norfolfk, UK). We considered maximum quantum yield of PSII (F_v_/F_m_; where F_v_ = [F_m_ - F_0_], F_m_ = maximum fluorescence yield, and F_0_ = minimum fluorescence yield) as a proxy of physiological performance (see, 18). One fully-developed attached leaf from each individual was dark adapted for 30 min (to obtain open PSII centers) using leaf-clips to ensure maximum photochemical efficiency. The fiber-optic and its adaptor were fixed to a ring located over the clip at approximately 10 mm from the sample and the different light pulses were applied (see below). Signal recordings and calculations were performed using the data analysis and control software provided with the instrument. Minimal fluorescence (F_0_) with all PSII reactions in the open state was determined by applying a weak modulated light (0.4 mol m^-2^ s^-1^). Maximal fluorescence (F_m_) with all PSII reaction centers in the closed state was induced by a 0.8s saturating pulse of white light (9000 µmol m^-2^ s^-1^). After 15 s, the actinic light (180 µmol m^-2^ s^-1^) was turned on and the same saturating pulse described previously was applied every 60s, until steady-state photosynthesis was reached in order to obtain the steady-state fluorescence yield (F_s_) and light-adapted fluorescence maximum (F_m_'). Finally, F_0_' was measured after turning the actinic light off and applying a 2 s far red light pulse (see, 18]).

At the end of the experiment we recorded the number of plants reaching anthesis and the number of capitula produced by each plant. Each capitulum was bagged with a transparent nylon mesh to prevent pollinator visitation and seed loss, and then seed production per capitulum was determined. Seed output was calculated as the ratio between the number of filled seeds and the total number of seeds (i.e., including aborted and predated seeds) produced per capitula (see, 26). This way, we obtained physiological performance and a fitness estimate for each individual.

### Statistical analysis

To test the hypothesis that seed-output is directly affected by physiological performance, we used standard Pearson correlation in order to investigate the relationship between quantum yield and seed output in *T. officinale* individuals from different localities. Each pair of measurements used to estimate Pearson correlations (physiological performance and seed-output) were recorded in the same individual. On the other hand, we assessed the R-square obtained from Pearson correlations for each locality with the precipitation seasonality (considered as the coefficient of variation of precipitations (CV = Standard deviation / Mean)) in order to evaluate if the strength of the physiology-fitness relationship is modified with environmental scenario. Finally, in order to evaluate if the relationship between physiological performance and seed-output of different localities had an adaptive value, we plotted the variation in fitness (seed output) of all *T. officinale* localities with the physiological performance (Fv / Fm) and precipitation seasonality in both homogeneous and heterogeneous watering condition. Climatic data from each sampled locality was downloaded from the WorldClim data-base (http://www.worldclim.org/) on the accumulated annual precipitation (rainfall, in mm), and rainfall seasonality (RS: standard deviation of the mean monthly rainfall, in mm).

## Results

**Figure 2:**
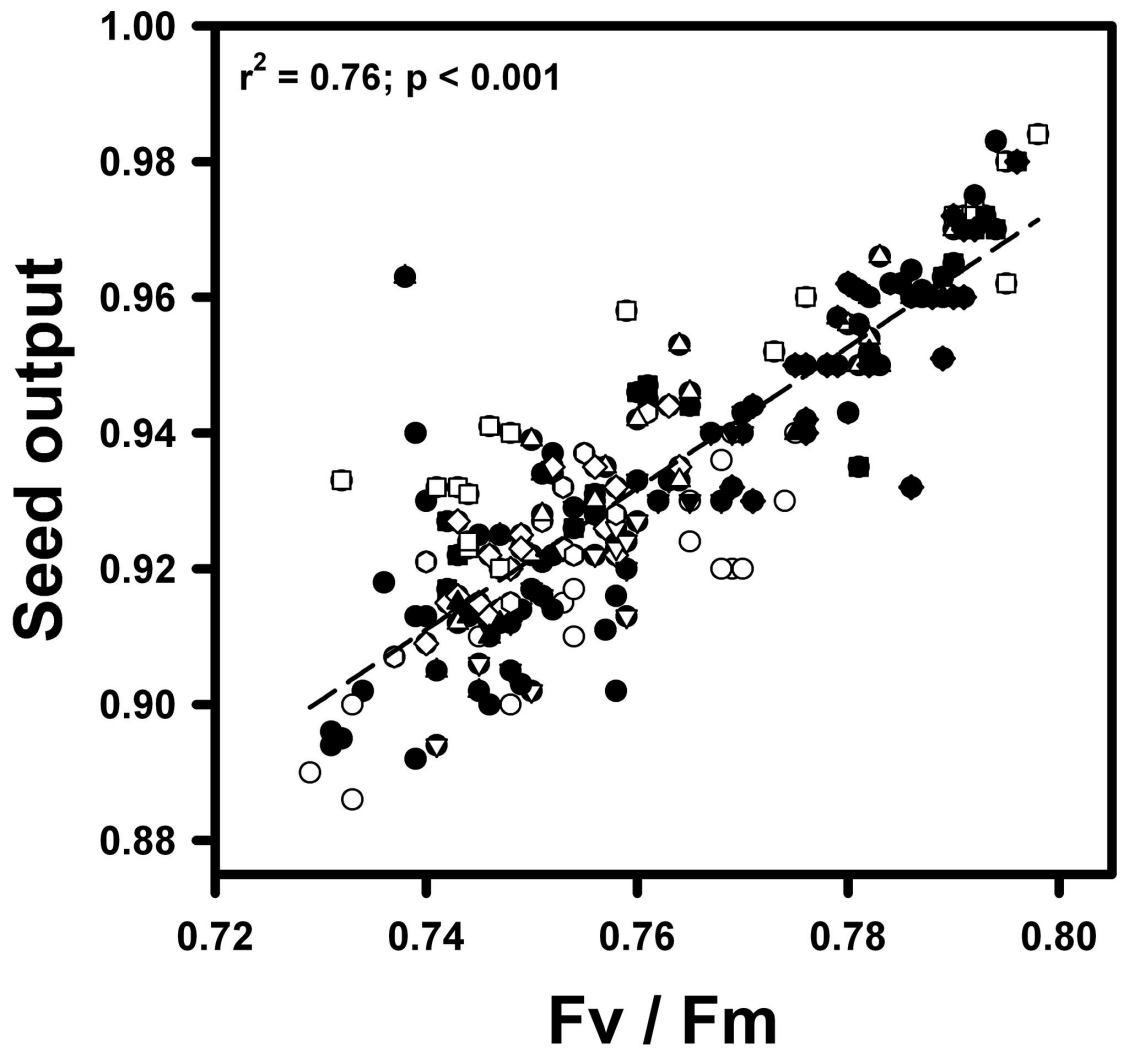
Relationship between maximum quantum yield (Fv / Fm) and seed output for 12 *Taraxacum officinale* individuals from 17 different localities distributed at worldwide. Combination of shape and color indicates different localities (total n = 204 individuals).

**Figure 3:**
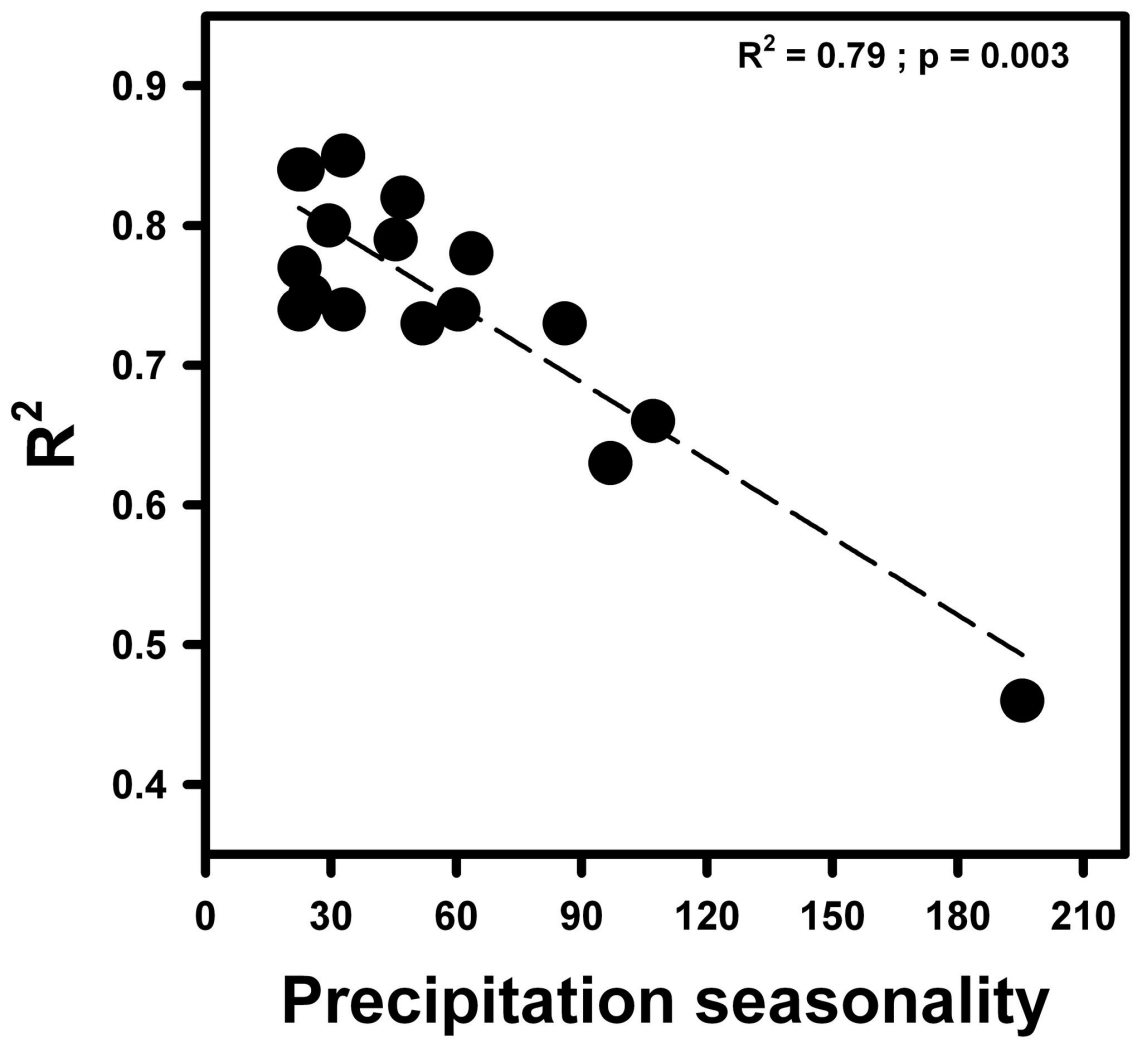
Pearson correlation between adjusted R-square and precipitation seasonality in 17 different populations of *Taraxacum officinale* distributed worldwide.

Overall, *T. officinale* across all localities showed a significant positive correlation between maximum quantum efficiency of photosystem II (Fv / Fm) and seed output (r^2^ = 0.76, p < 0.001; Figure 2). In addition, these correlations registered the lower and higher values in individuals from Trujillo, Perú and París (France) locality (r^2^ = 0.46 and r^2^ = 0.85, respectively). On the other hand, there was a significant negative correlation (r^2^ = 0.79, p = 0.033) between the adjusted R-square obtained from Pearson correlations in each locality and environmental heterogeneity (Table 1, Figure 3).

**Figure 4:**
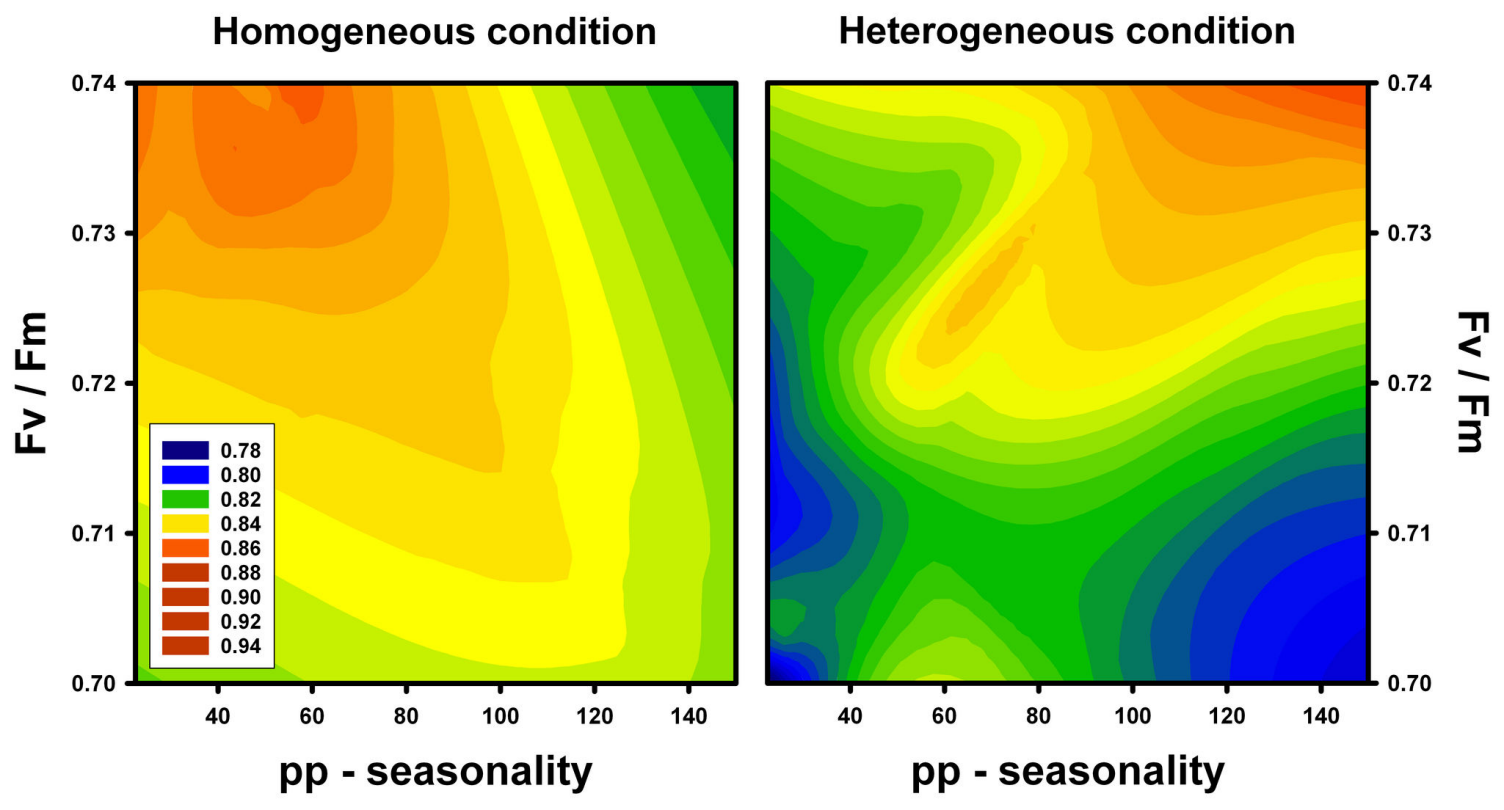
Relationship between fitness (seed-output) and maximum quantum yield (Fv / Fm) of *Taraxacum officinale* from localities with different precipitation seasonality (considered as the coefficient of variation of precipitations (CV = Standard deviation / Mean)), and exposed to different irrigation treatments. Seed-output was calculated as the ratio between the number of filled seeds and the total number of seeds (i.e., including aborted seeds) produced per capitula. Seed-output is showed with different colors as indicated in the box inside of the figure.

In both homogeneous and heterogeneous watering conditions, seed-output was greater in individuals with higher values of physiological performance (Figure 4). Under the homogeneous regime of watering, those individuals with higher maximum quantum efficiency (Fv / Fm) and from localities with low precipitation seasonality showed a greater seed-output. Contrarily, under a heterogeneous watering regime, those individuals of *T. officinale* with higher values of Fv / Fm and from localities with high precipitation seasonality showed a greater seed-output (Figure 4), suggesting adaptive value in each environmental condition.

## Discussion

Despite the widely accepted relationship between environmental stress and fitness, and chlorophyll fluorescence as an indicator of environmental stress [1,4,19,20], a link between the chlorophyll fluorescence and fitness has been not clearly demonstrated. Plants of *T. officinale* that showed higher maximum quantum efficiency of Photosystem II produced higher seed-output, suggesting that physiological performance is a good predictor for fitness. Similar results have been found in the past. For example, Figueroa et al. (1997) [19] showed that those Mediterranean species with higher survival percentage showed higher physiological performance towards end of the growing season. These results could be attributed to changes in photosynthetic pathways, reducing the specificity factor of Rubisco from carboxylation toward oxygenation, with a decrease in the photosynthetic performance. On the other hand, thereduction of the net photosynthetic CO_2_ assimilation rate has been attributed to stomatal closure and increased diffusive resistance of the mesophyll cells, the main cause for decreased photochemical efficiency under fluctuating water availability [3,27-29]. Additionally, a decrease in Fv / Fm under fluctuating water availability could reflect an alteration in flow of electron transporter rate in photosystem II (PSII) that reduces the efficiency of photosynthesis [30].

Under water shortage, as it is expected mainly in localities with highly seasonal precipitation, down-regulation of photosynthesis or photoinhibition cause overexcitation of the photochemical system [31-33]. Thus, a high concentration of reduced electron acceptors may increase the regeneration of free radical molecules, which can damage PSII components, even the thylakoid membrane, negatively affecting physiological performance [30,34,35]. Free-radical damage to the photosystems can potentially have a whole-plant effect and negatively impact fitness, although we not measured the effect of free-radicals, negative effects that decrease the fitness in *T. officinale* is not rule out here. There is a strong relation between PSII efficiency and CO_2_ assimilation. Some authors have argued that different fluorescence parameters could be appropriate estimators of plant performance and therefore positively linked with plant fitness [13,36]. Our results support the idea that maximum quantum efficiency of Photosystem II is positively related to fitness in *T. officinale* individuals, and rainfall seasonality can drive the strength of this relationship. Thus, we suggest that a rapid measurement of chlorophyll fluorescence parameters would be used to estimate the potential fitness, at least of the invasive *T. officinale*.

On the other hand, adjusted R-square and precipitation seasonality were negatively correlated reflecting an interesting trade-off between environmental stability and reproductive maternal fitness. This could be a pattern in invasive plant species. For example, several studies suggested that under heterogeneous or stressful conditions some invasive plants can maintain a relative positive fitness (as shown here for *T. officinale*), but under homogeneous or resource-rich conditions they can take a larger advantage through greater fitness [37-39]. In this sense, Richards et al. (2006) [37] suggest that invasive plants can modify its ecophysiological traits in order to maintain a constant fitness in stressful or heterogeneous climatic conditions, and maximize its fitness under favorable or homogeneous conditions. This characteristic present in several invasive plant species is called the “Jack-and-Master” strategy and help to invade environments with different climatic conditions. Considering that *T. officinale* allocated high amount of resources to seeds in both heterogeneous and homogeneous water conditions, the “Jack-and-Master” strategy could be considered as one of the main strategies used by this species to invade broad areas worldwide. An interesting finding was that the relationship between physiological efficiency and seed-output was not depend on the environmental context, but was affected by origin of each individual. In both homogeneous and heterogeneous regimes, plant fitness was more strongly related to fluorescence parameters based on the geographic origin of individuals, suggesting the presence of an adaptive value in this relationship. This implies that the usefulness of chlorophyll fluorescence parameters as estimators of physiological performance is maintained under different environmental conditions. Nevertheless, the strength of this relationship in each environmental scenario depends of the precipitation seasonality where each individual is originated, suggesting an incipient evolution in the physiological performance-fitness relationship. Instead, under heterogeneous watering conditions, *T. officinale* individuals originated from localities with seasonal rainfall showed a stronger relationship between physiological performance and fitness. Contrarily, under homogeneous water conditions, a stronger relationship between physiological performance and fitness were found in those individuals from locations of low rainfall seasonality. Thus, we can suggest that *T. officinale* individuals have been subjected to different environmental regimes that have determined local adaptation at least in terms of maternal fitness. On the other hand, those *T. officinale* individuals exposed to water conditions different from those observed in its original location showed a reduced capacity to assimilate carbon. This mismatch may decouple the linear relationship between PSII efficiency and carbon fixation due to changes in the rate of photorespiration or pseudocyclic electron transport [40]. Thus, in those individuals subjected to environmental conditions different to those where they were originated, more electrons must be transported trough PSII for each molecule of CO_2_ assimilated in relation to those individuals habiting environments close to those where were originated. This adaptive response based on high values of physiological performance and seed-output, and can be translated in whole performance gains if we consider all the study genotypes of *T. officinale* as a whole species more than individuals from different geographic localities.

In conclusion, our results suggest that the maximum quantum yield of PSII (Fv / Fm) seems to be a good predictor for plant fitness, mainly under homogeneous conditions, because even minor changes in plant physiological status can have direct and significant consequences for plant reproduction. Considering that drought stress is considered as one of the main constraints to plant survival, growth and reproduction [5], rapid measurements as those obtained from chlorophyll fluorescence could give a relatively fast estimate about of individual’s fitness in changing environments.
